# Recent Advances in Toll Like Receptor-Targeting Glycoconjugate Vaccines

**DOI:** 10.3390/molecules23071583

**Published:** 2018-06-29

**Authors:** Qingjiang Li, Zhongwu Guo

**Affiliations:** Department of Chemistry, University of Florida, 214 Leigh Hall, Gainesville, FL 32611, USA; qingjiang.li@chem.ufl.edu

**Keywords:** conjugate vaccine, carrier molecule, adjuvant, toll-like receptor ligand, lipopeptide, monophosphoryl lipid A

## Abstract

Many malignant cell surface carbohydrates resulting from abnormal glycosylation patterns of certain diseases can serve as antigens for the development of vaccines against these diseases. However, carbohydrate antigens are usually poorly immunogenic by themselves, thus they need to be covalently coupled with immunologically active carrier molecules to be functional. The most well established and commonly used carriers are proteins. In recent years, the use of toll-like receptor (TLR) ligands to formulate glycoconjugate vaccines has gained significant attention because TLR ligands can serve not only as carrier molecules but also as built-in adjuvants to form fully synthetic and self-adjuvanting conjugate vaccines, which have several advantages over carbohydrate-protein conjugates and formulated mixtures with external adjuvants. This article reviews recent progresses in the development of conjugate vaccines based on TLR ligands. Two major classes of TLR ligands, lipopeptides and lipid A derivatives will be covered with more focus on monophosohoryl lipid A (MPLA) and related analogs, which are TLR4 ligands demonstrated to be able to provoke T cell-dependent, adaptive immune responses. Corresponding conjugate vaccines have shown promising application potentials to multiple diseases including cancer.

## 1. Introduction

Cells are covered by a dense glycocalyx that is mainly comprised of glycoproteins and glycolipids. The carbohydrate motifs, carrying signaling information, are involved in cell recognition and responses. Each cell uses these signals to mediate communication, proliferation, survival and various other cellular activities [1,2,3]. The human immune system also relies on the recognition of these signaling molecules to differentiate invasive cells from native ones. When presented to the immune system in the proper ways, surface carbohydrates of foreign cells can serve as antigens to induce immune responses that eradicate the invasive cells. Thus, cell surface carbohydrates are ideal candidate antigens for vaccine development. In this context, however, a major caveat is that carbohydrate antigens are typically weakly immunogenic [4,5,6]. In addition, unlike protein antigens, which usually elicit immune responses via T cell-dependent pathways [7], carbohydrate antigens alone can only induce innate immune responses and fail to induce T cell-dependent immunity [4,8]. Thus, an ultimate goal of carbohydrate-based vaccine development is to achieve optimized presentation of carbohydrate antigens and switch of innate immunity against them to adaptive immunity. Covalent coupling of carbohydrates to carrier molecules or adjuvants, which are proteins, lipids or other molecules having immunological stimulating activities, to form functional glycoconjugate vaccines has proved to be a solution to the problem. For example, conjugation with an adjuvant can alter the immune responses to carbohydrates into T cell-dependent immunities that can have improved therapeutic efficacy and lasting immunological memory [9,10,11]. 

### 1.1. Conjugate Vaccines

The first generation of vaccines were attenuated live viruses and bacteria, which were often either insufficient or still infectious/inflammatory due to inherit difficulties in the attenuation process. Vaccine formulations consisting of purified ingredients, including antigens and adjuvants, have provided a better solution since these vaccines can induce more specific immune responses and reduce undesired activity. Accordingly, many external adjuvants, including mineral salts, liposome emulsions, Freund’s adjuvant, protein toxins or small molecules such as toll-like receptor (TLR) ligands, have been developed to probe and augment the activities of experimental and clinical vaccines. Although various adjuvants may have different mechanisms of action, their general objective aims at increasing the uptake of target antigens by antigen presenting cells (APCs), such as dendritic cells (DCs) and macrophages, activating APCs, and thereby triggering immunoregulatory cytokine production and a cascade of immune responses including T cell recruitment. However, many adjuvants are derived from bacteria/virus and may bring about side effects and toxicity similar to that of attenuated live cell vaccines. 

An alternative approach is to covalently link antigens to an immunostimulating carrier molecule or adjuvant to construct conjugate vaccines that have proved to have advantages over heterogeneous antigen/adjuvant mixtures [12,13]. First, by incorporating an immunostimulating moiety, the resultant conjugate vaccines become more immunogenic. Second, conjugate vaccines have relatively defined compositions. Third, the toxicity of carrier molecules or adjuvants derived from natural sources may be reduced upon conjugation with large hydrophilic carbohydrate antigens.

Originally proteins were used as carrier molecules in conjugate vaccine development. In 1929, Avery et al. conjugated a polysaccharide to a protein carrier to create the first conjugate vaccine with enhanced immunogenicity [14,15,16]. Since then, numerous bacterium-derived proteins, such as tetanus toxoid (TT), diphtheria toxoid (DT) and meningococcus B outer membrane protein complex (OMPC), and other proteins, such as keyhole limpet hemocyanin (KLH), have been used to formulate carbohydrate-based conjugate vaccines [17,18]. These protein carriers have witnessed great success. For example, a series of glycoconjugate vaccines against various infectious diseases, such as *H. influenzae* type B, *Neisseria meningitidis* and pneumonia, have been licensed for clinical use [19] and several anticancer glycoconjugates vaccines have entered clinical trials. Mechanistic studies revealed that T cells can recognize the sugar epitopes of glycoconjugate vaccines in the context of major histocompatibility complex (MHC), allowing the binding of sugar epitopes to MHC and activation of the adaptive immune system [20]. However, most protein carriers are very immunogenic themselves, thereby complicating the evaluation of immunogenicity against the antigen. In addition, conjugation sites and equivalents of carbohydrate antigen to carrier protein are random and uncontrollable, resulting in inconsistent efficacy from batch to batch. This has led to the exploration of conjugate vaccines having small molecule carriers or adjuvants, such as ligands to specific receptors like TLRs, mannose receptors and lectin receptors on dendritic cells (DCs) and other APCs. 

### 1.2. Toll Like Receptors

The TLR family is a class of pattern recognition receptors (PRRs) of mammalian species, which are considered the bridge between innate and adaptive immune responses [21]. Facing the daunting task of fending off ever evolving microbes with tremendous molecular diversity and heterogeneity, mammalian species have developed the PRR-centered pattern-recognition strategy to detect the conserved molecular patterns of characteristic microbes. As the best representative of PRR functions, TLRs detect pathogen-associated molecular patterns (PAMPs) of invading cells and trigger the first level of defense, the innate immunity. TLRs on dendritic cells are also involved in the initiation of adaptive immune responses and play a key role in bridging innate and adaptive immunities [21]. Thus far, ten human TLRs and two TLR signaling pathways have been identified. The predominant myeloid differentiation primary response 88 (MyD88)-dependent pathway is used by TLR1, 2 and 4–9 [22], while the MyD88-independent pathway is only used by TLR3 and 4 [23,24]. TLR1, 2, 4, 5 and 6 are expressed on the cell surface whereas TLR3, 4, 7, 8 and 9 are in endosomal compartments. Some of them form complexes when binding to ligands to be activated. TLR2 can form complexes with either TLR1 or TLR6, and TLR4 associates with myeloid differentiation factor 2 (MD2) [25] after being activated by lipopolysaccharide (LPS) ligand [26]. Each of these TLRs or their complexes recognize and are activated by multiple molecular patterns characteristic of bacteria or viruses. For example, TLR1 can be activated by multiple triacyl lipopeptides; TLR2 recognizes various glycolipids, lipopeptides and lipoteichoic acid (LTA); TLR3 recognizes double strand RNA; TLR4 can be activated by LPS, some heat shock proteins and heparan sulfate fragments; TLR7 and 8 can be activated by multiple small organic molecules and single strand RNAs.

The ligands of all ten human TLRs have been identified and extensively examined, paving the path for their applications to immunological studies and vaccine development. The well-defined activation mechanisms and signaling pathways of TLRs represent a major advantage of TLR ligands over proteins as carrier molecules for formulating glycoconjugate vaccines as this allows for rational design and precise structural modification towards optimized immunostimulating property. As a result, many TLR ligands, such as tripalmitoylated/dipalmitoylated cysteine (Pam_3_Cys/Pam_2_Cys), lipid A analogues, recombinant flagellin and imidazoquinoline analogues, have been covalently conjugated with antigens and evaluated as vaccine carriers. In fact, the ligands of all 9 TLRs, except for TLR10 which is believed to be a negative regulator of TLR signaling [27], have been probed as conjugate vaccine carriers/adjuvants. It has been demonstrated that the defined structures of conjugate vaccines with TLR ligands as carrier molecules have facilitated structure-activity relationship studies, which is critical for tuning and optimizing the structure of carrier molecules. In addition, the on-target delivery of antigens to dendritic cells can further enhance their uptake and avoid adjuvant overuse and thereby minimize the endotoxicity. 

Although numerous TLR ligands have been identified as immunostimulants, some of which were discovered even long before TLRs, only a handful of them have been successfully applied to conjugate vaccine development. Some ligands, such as lipoteichoic acids, are limited by the accessibility due to their complex structures. Some ligands, such as small heterocycles, are excluded because the target TLRs locate in cell compartments. Others, such as RNAs, although commercially available, are limited by the intrinsically poor drug properties, including difficult characterization and analysis after conjugation. Two classes of TLR ligands, lipopeptides and lipooligosaccharides, stand out from the rest because of their outstanding potential as built-in adjuvants for carbohydrate-based conjugate vaccines. Aside from these two major classes of ligands, other TLR ligands such as Flagellin (an agonist of TLR5), small heterocycle molecules (agonists of TLR7/8) and unmethylated cytidine-phosphate-guanosine (CpG) oligodeoxynucleotides (agonists of TLR9) have also been studied as built-in vaccine adjuvants. Most of these compounds are, however, linked to protein or peptide antigens and have been reviewed elsewhere [26,28], and conjugation of these ligands with carbohydrate antigen has not been reported.

This mini-review will only cover TLR ligands employed in conjugating with carbohydrate antigens to formulate glycoconjugate vaccines, with an emphasis on the optimization of monophosphoryl lipid A (MPLA) as a vaccine carrier. Moreover, we choose to limit its scope to carbohydrate-TLR ligand conjugates, TLR ligand conjugates with peptide or protein antigens are not discussed either.

## 2. Lipopeptides as Carrier Molecules or Adjuvants for Vaccine Development

Lipopeptides as agonists of TLR1, 2, 6 and 10 were the first and most studied TLR ligands applied to glycoconjugate vaccine development. Pam_3_Cys and its tetralysine analog Pam_3_CSK_4_ were demonstrated to activate TLR1/TLR2 heterodimers [29], and Pam_2_Cys and Pam_2_CSK_4_ were shown to be potent agonists of TLR2/TLR6 heterodimers [30]. Other ligands of TLR2 or TLR2/TLR6 heterodimers include lipoaminoacids and macrophage-activating lipopeptide 2 (MALP-2). All of these lipopeptides and lipoaminoacids have been exploited as built-in adjuvants to conjugate with carbohydrate or glycopeptide antigens for conjugate vaccine development. 

**Scheme 1:**
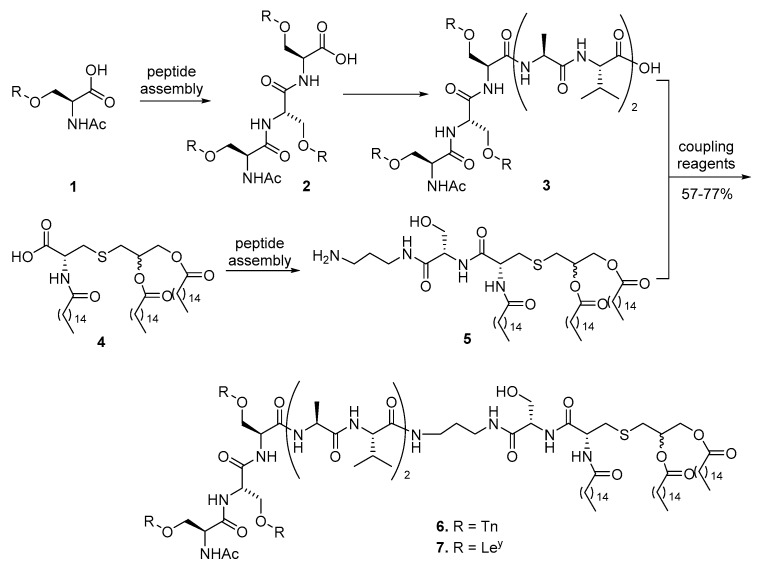
Synthesis of trimeric glycopeptide conjugate vaccines with Pam_3_Cys as the carrier molecule.

Pam_2_Cys, introduced by Hopp even before the discovery of TLRs [31], was the first lipopeptide used for conjugate vaccine development, while the report by Jung et al. [32] had described both Pam_3_Cys and Pam_2_Cys. Since then, Pam_3_Cys, Pam_2_Cys, and their derivatives have become the most studied lipopeptide vaccine carrier because of their defined structures and commercial availability. In 1994, tumor-associated carbohydrate antigen (TACA) Tn was covalently conjugated to Pam_3_Cys by the Toyokuni group to construct dimeric and trimeric vaccine **6** (Scheme 1) [33,34]. First, glycopeptide **3** containing three Tn antigens was assembled by standard peptide synthesis method, and its terminal acid was then converted into an active ester to be linked to Pam_3_Cys **5** with an amide bond in a 57% yield. A robust immune response against this conjugate was observed without an external adjuvant. IgG antibody was observed, although in low concentration, indicating the recruitment of T cells and initiation of adaptive immune response. This was the first experimental evidence and proof-of-concept for the self-adjuvanting property of glycoconjugate vaccines with TLR ligands as carrier molecules. Inspired by this research, Danishefsky and coworkers synthesized blood type antigen Le^y^, a hexasaccharide, and linked it to the same carrier to obtain conjugate **7** (Scheme 1), which also elicited strong immunoglobulin M (IgM) type of immune response [35,36]. Using the same strategy, the Danishefsky group synthesized a pentavalent vaccine, with each of Tn, sTn, TF, Le^Y^ and Globo H antigen conjugated to Pam_3_Cys [37]. However, no immunological data was reported for this amazingly complex glycoconjugate.

**Scheme 2:**
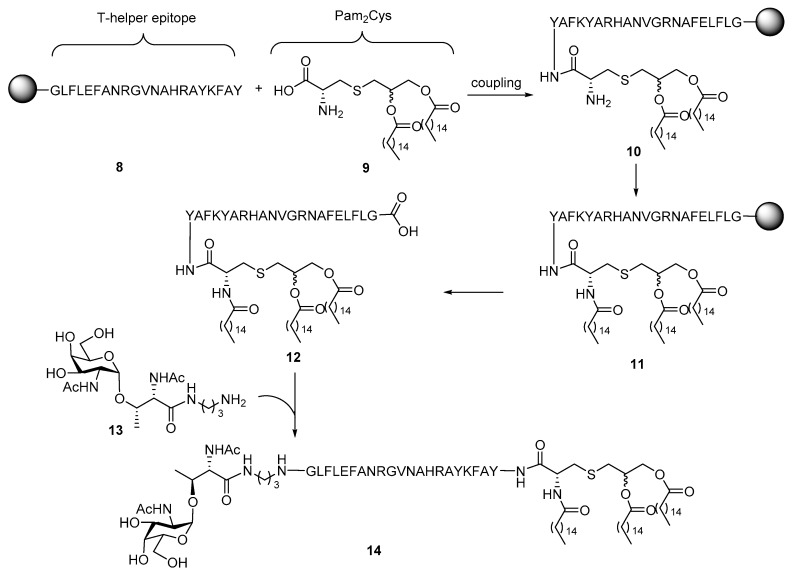
Synthesis of a three-component conjugate vaccine containing a Tn antigen, the Pam_3_Cys epitope, and a helper T cell epitope.

Unfortunately, none of the above glycoconjugates of Pam_3_Cys elicited robust IgG antibody response, which is considered the character of adaptive immunity. To achieve immunity class switch and induce IgG antibody production, the Boons group designed and synthesized a three-component conjugate vaccine **14** (Scheme 2) [38]. In addition to the TLR1/2 ligand Pam_3_Cys as built-in adjuvant and Tn antigen as the B cell epitope, a helper T cell epitope derived from *Neisseria meningitis* was also incorporated in the conjugates as the third component. A helper T cell epitope was believed to activate helper T cells and achieve class switch of antibodies [39]. As shown in Scheme 2, T-helper epitope peptide **8** was coupled with Pam_2_Cys **9** on a solid support. Conversion of Pam_2_Cys to Pam_3_Cys was performed after coupling because the low reactivity of Pam_3_Cys, due to its high hydrophobicity, with peptides. After introduction of the third lipid chain and dissociation from the beads, Tn antigen **13** with terminal amine was linked to lipopeptide **12** to complete the synthesis. Although only low to moderate IgG antibody titer was elicited in the subsequent immunological studies of **14** in mice, this is the first proof-of-concept that incorporation of T-helper epitope to facilitate the antibody class switch in conjugate vaccines. 

**Scheme 3:**
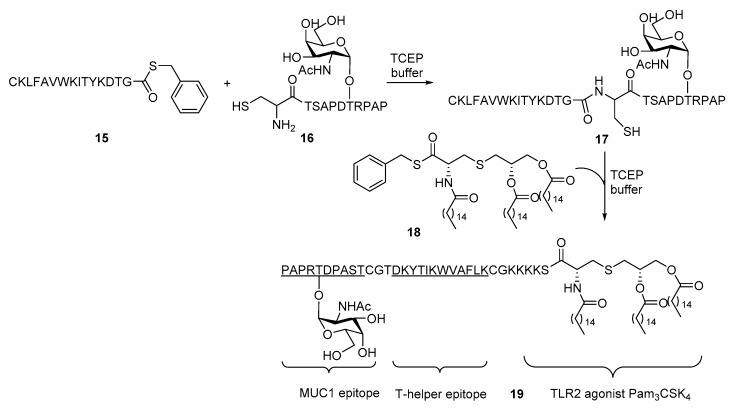
Synthesis of MUC1 glycopetide-based three-component conjugate vaccine **19** containing the Pam_3_CSK_4_ epitope and a helper T cell epitope.

Despite the promising immunological results of the above three-component conjugate vaccine, new synthetic challenges of covalently linking glycopeptide to Pam_3_Cys cast a shadow over the application of this strategy. In the synthesis of **6** and **7**, Pam_3_Cys could be directly linked to the glycopeptide antigen N-terminus. However, with the increase of complexity of glycopeptide moieties by introducing the helper T cell epitope, their coupling with the lipopeptide carrier has become more difficult. Many approaches to achieve glycopeptides conjugation with Pam_3_Cys, including preinstallation of molecular handles such as alkyne or active ester, in the Pam_3_Cys moiety have been described [40,41,42,43,44,45]. In the Boons group, native chemical ligation (NCL) was brought into the synthesis of three-component vaccine **19** (Scheme 3) [46,47]. The reaction between the thioester of helper T cell epitope **15** from polio virus and tumor-associate antigen **16** derived from cell surface associated Mucin 1 (MUC1) with a terminal cysteine in the presence of tris(2-carboxyethyl)phosphine (TCEP) efficiently constructed the amide linkage between the two moieties. The N-terminal of helper T cell epitope **15** was also modified with a cysteine. Similarly, the reaction between the thioester of TLR1/2 ligand Pam_3_CSK_4_ **18** with the terminal cysteine in **17** furnished the three-component conjugate vaccine **19**. It was later discovered that the NCL step could be further improved by embedding the thioester of Pam_3_CSK_4_ in liposomes [48]. In addition to the improvement from a synthetic perspective, conjugate **19** also induced much improved immune response with over 100 folds of IgG antibody titers against MUC1 as compared to **14**. The increased potency of **19** in the immunological study undoubtedly demonstrated the importance of vaccine design. It is noteworthy that enantiomerically pure glycerol was used to prepare Pam_3_CSK_4_ in contrast to previous examples. However, a later study by Shi et al. [49] demonstrated that the absolute configuration had no significant impact on the immunogenicity, which is in consistent with the earlier study on their peptide conjugates [50].

**Scheme 4:**
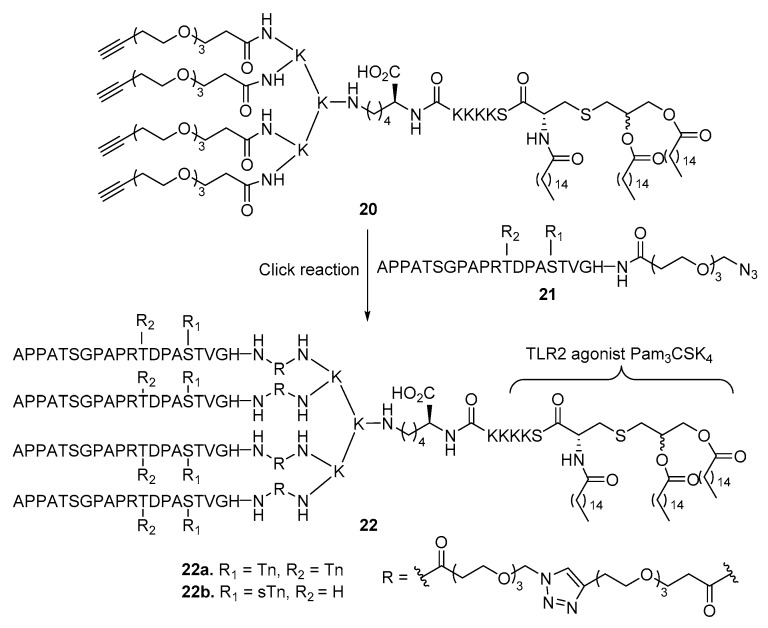
Synthesis of tetravalent MUC1 glycopeptide-based vaccines with Pam_3_CSK_4_ as the carrier molecule or adjuvant.

In pursuing a better presentation of antigens to the immune system, Cai et al. [51] synthesized cluster conjugate vaccines that had four MUC1 glycopeptide antigens linked to Pam_3_CSK_4_ (Scheme 4). A cluster effect showing positive correlation between the antigen valency and the antibody production was clearly demonstrated. In addition, stronger binding and higher cytotoxicity of the antiserum to cancer cells were also observed with the tetravalent glycopeptide antigen vaccines **22a** and **22b** as compared to the mono- and divalent counterparts.

**Scheme 5:**
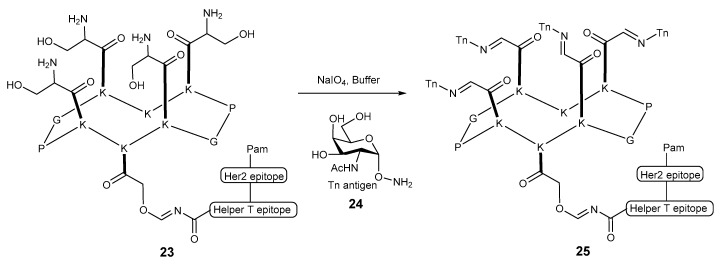
Synthesis of tetravalent glycopeptide conjugate vaccine **25** based on RAFT.

In another report, the Dumy group [52,53] loaded multiple Tn antigens onto a cyclic decapeptide scaffold using a strategy called regioselectively addressable functionalized template (RAFT) developed by the same group earlier [54]. When constructing this peptide scaffold, versatile handles were introduced to lysine so that subsequent conjugation with the Tn antigen could be achieved in aqueous environment (Scheme 5). After excellent IgG immune response was observed for conjugate **25**, a systematic biological study was performed. Two conjugates with either linear or branched palmitoyl-peptide were evaluated in several aspects including the uptake, processing, cross-presentation pathways and how the B and T cell immunogenicity were affected. While both conjugates inhibit tumor growth, they appear to undergo different cross-presentation pathways. The linear molecule proved to be superior in terms of activating dendritic cells and inducing potent TLR2-dependent T cell activation. 

**Scheme 6:**
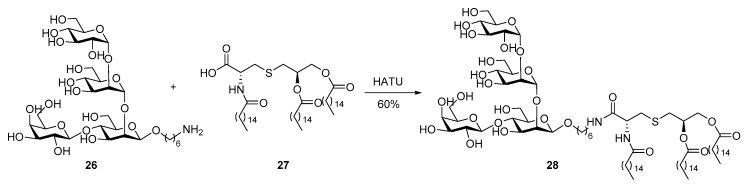
Synthesis of a carbohydrate-based Leishmaniasis vaccine with Pam_3_Cys as the carrier molecule.

Direct linkage of oligosaccharide antigens to Pam3Cys to form synthetic vaccines was also reported. In 2001, the Seeberger group [55] conjugated a leishmaniasis tetrasaccharide antigen with Pam_3_Cys via an amide bond (Scheme 6). However, no immunological data was provided, thus we have no idea about the efficiency of this vaccine to induce immune responses in vivo.

The less lipidated lipopeptide Pam_2_Cys is a motif derived from MALP-2 of mycoplasma, which lacks the cell wall [56], and was shown to activate TLR2/6 heterodimer [30]. Compared to their Pam_3_ counterparts, Pam_2_Cys and Pam_2_CSK_4_ are more soluble in aqueous solutions, which is considered a significant pharmacological advantage as it facilitates the trafficking of vaccines. Moreover, the free amino group of Pam_2_Cys allows for further modification, which may add unique functions to the conjugates. For example, in a recent study on cancer imaging conducted by the Vagner group, a fluorescent tag or metal chelate was attached to the amino group through a PEG spacer without affecting the potency of Pam_2_Cys [57]. Despite these advantages, Pam_2_Cys and Pam_2_CSK_4_ are believed to be more suitable for conjugation with peptide antigens because their conjugates with carbohydrate antigens were not satisfactory and less studied [46].

**Scheme 7:**
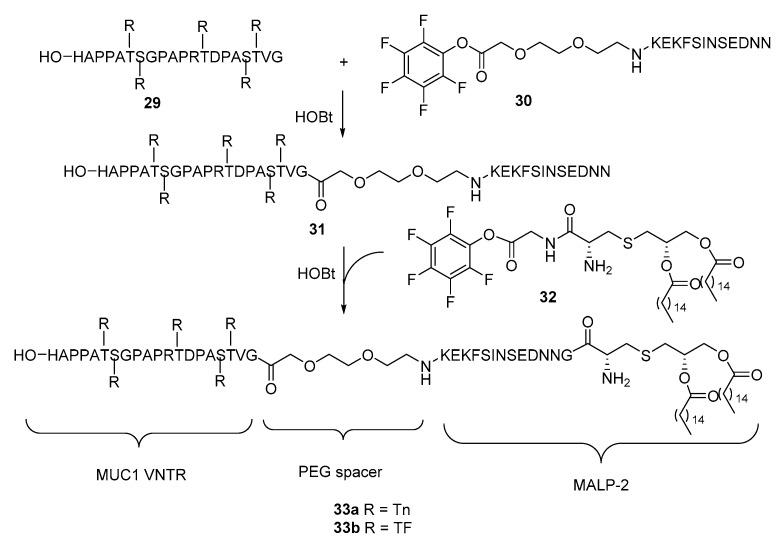
Synthesis of MUC1 glycopeptide-MALP-2 conjugates **33a** and **33b** as vaccine candidates.

McDonald et al. [58] synthesized the complete MALP-2 and conjugated it with the extracellular domain of MUC1 consisting of variable-number tandem repeat (VNTR) sequence with twenty amino acids and five *O*-glycans (Scheme 7). A fragment condensation strategy developed by Payne et al. [42,59,60] to construct MUC1-Pam_3_Cys conjugates was adopted for the synthesis of target molecule **33**. MUC1 glycopeptide **29** and active ester of PEG-peptide **30**, which were both obtained from solid-phase peptide synthesis (SPPS), were coupled followed by conjugation with Pam_2_Cys via active ester **32**. Immunological studies of the resultant glycoconjugates **33a** and **33b** showed that they induced the production of several isotypes of IgG antibodies. The production of IgG antibody suggests that activation of TLRs by lipopeptides are sufficient and the T cell epitope may not always be necessary to initiate adaptive immune response [61]. It was thus possible that the failure of the aforementioned vaccines **6** and **7** to elicit IgG antibody production might be ascribed to other reasons, such as inefficient presentation of TLR ligands and lack of spacing between functional moieties. It is believed that an immunologically silent spacer like ethylene glycol is necessary to diminish the interplay between MUC1 antigen and peptide of Pam_3_Cys analogs [62]. In fact, strong interactions between carbohydrate and peptide [63] affect the MUC1 antigen confirmation [64,65], resulting in a particular 3-D structure that can be recognized by immune cells as an entity. Disturbance from Pam_3_Cys peptide may cause change in the confirmation of MUC1. 

Pam_3_Cys, Pam_2_Cys, and MALP-2 are among the most studied TLR ligands as adjuvants and their structure-activity relationship (SAR) studies have been performed extensively in the attempt to improve their adjuvant properties. Optimization towards higher binding affinity with TLRs or their heterodimers and improvement of the pharmacodynamics properties are the two major directions of the SAR studies. The crystal structure of a TLR1/2 heterodimer has shown that the stereochemistry and orientation of acyl chains in the lipopeptide ligands are critical for their optimal fitting in the hydrophobic pocket of TLR1/2 heterodimer [66]. Therefore, the stereochemistry, length, and number of acyl chains in the lipopeptides were investigated in correlation with their agonist and adjuvant properties [61,67,68,69]. For example, it was disclosed that switching the amide linkage of lipid chains in Pam_3_Cys to a urea linkage was beneficial for DC maturation [70] and replacing the two ester linkages with amide bonds could improve its stability towards esterases [71]. The position of Pam_3_Cys in the conjugate vaccine was also found to be related to the biophysics of the conjugates [72]. When adjuvant was placed in the middle of B and T epitopes, the conjugate vaccine was found to be most soluble and immunogenic. In another study to develop new TLR ligand adjuvants, lipoamino acids with various lipid chains were synthesized and evaluated against Pam_3_CSK_4_ in three-component conjugate vaccines by the Toth group and they found that two repeating units of lipoamino acids with 16 carbon lipid chains were most immunostimulating by activating TLR2, although not as potent as Pam_3_CSK_4_ [73]. These investigations have been reviewed recently [74,75] and will not be elaborated here. 

As amphiphiles, the above lipopeptides form micelles in aqueous environments. Micelle formation can improve the in vivo stability and presentation of antigens to B cells, and more importantly, the proper particle size (5–100 nm) is critical for passive drainage of micelles into lymph nodes where naïve T and B cells reside [12]. The assembly of three-component MUC1-T epitope-Pam_3_Cys conjugates in an aqueous environment were evaluated by the Payne group [60], who found that a micelle size of 17–25 nm is ideal for passive drainage to lymph nodes. 

## 3. MPLAs as Carrier Molecules or Adjuvants for Vaccine Development

### 3.1. TLR4 Ligands as Adjuvants

Among all TLRs, TLR4 stands out due to several unique features of its signaling pathway. It was the first human TLR discovered [76] and thus the most studied. It forms a complex with MD2 for action [25] and signals through the MyD88-dependent pathway on the cell surface. The complex can be internalized to endosome, creating an additional activation site for TLR4 signaling. Internalized TLR4-MD2 complex triggers the toll/interleukin-1 receptor (TIR) domain-containing adapter-inducing interferon-β (TRIF) pathway of signaling [77], adding another profile that can be targeted. These features allow for unusual flexibility in attenuating ligand structures towards better adjuvant properties in conjugate vaccine. This is supported by the variety of TLR4 ligands, which range from LPSs [76] to proteins (heat shock proteins [78]), small molecules (opioid drugs [79]) and even metals (nickel [80]).

Among all TLR4 ligands, lipid A is the most famous and studied. It is lipophilic anchor and the main biologically active motif of LPS, which is a constituent of gram-negative bacteria cell wall. Small amounts of LPS/lipid A released from invading bacteria can serve as a “danger signal” for host immune systems to initiate counteracts, which may be reflected as inflammation. If not controlled, this can lead to fatal septic shock. 

**Figure 1:**
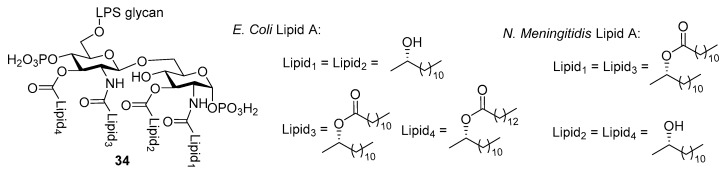
Conservative structure of lipid A as the core of LPS.

Depending on the bacterial strain where LPS is isolated, lipid A may vary in structure, especially in terms of the length, saturation, and number of fatty acid chains. However, a shared core structure of β-d-glucosamine (GlcN)-(1→6)-β-d-glucosamine disaccharide with 1,4’-di-*O*-phosphorylation and 2,2’-*N*-3,3’-*O*-acylation, is always observed with lipid A (Figure 1). Although lipid A has been known to be strongly immunostimulatory and evaluated as a vaccine adjuvant for decades, it’s utility was limited by the endotoxicity related to septic shock mentioned above. Fortunately, it was found that mild hydrolytic treatment of LPS, e.g., that from *Salmonella minnesota* R595, gave rise to monophosohoryl lipid A (MPLA) [81], which was 1000-fold less toxic with retained immunostimulatory property. This discovery ensures the safe use of MPLA and led to the prosperity of lipid A-related research. For example, the combination of safety and efficacy with the identification of TLR4 as the signaling receptor of lipid A has finally paved the way for the application of MPLA as a vaccine carrier.

### 3.2. Use of Lipid A Derivatives for Vaccine Development and/or as External Adjuvants

Since then, much effort has been focused on the immunostimulatory activities of MPLAs as external adjuvants and extensive clinical studies have been reported. In combination with aluminum salts or QS21 (also known as AS04 and AS02, respectively), a heterogenous mixture of MPLAs having varied fatty acids in length and acylation pattern was administered together with multiple antigens against various diseases. Vaccines against hepatitis B [82], HIV [83], malaria [84], and multiple cancers [85,86,87,88,89] have entered clinical trials. Inspired by the success of MPLA mixtures, lipid A mimetics, such as aminoalkyl glucosaminide 4-phosphates (AGPs), have been developed and probed as external adjuvants. After being extensively evaluated in human clinical trials, a few APGs have demonstrated to be an efficacious adjuvant with an excellent safety profile [90]. An AGP-based vaccine against Hepatitis B has been approved for use in Argentina [91]. Compared to MPLA, AGPs have much simplified structures, rendering their synthesis easier and more efficient while exhibiting immunostimulatory activity comparable to or better than MPLA in preclinical studies.

### 3.3. MPLA Analogs as Carrier Molecules and Built-in Adjuvants for Conjugate Vaccine Development

**Scheme 8:**
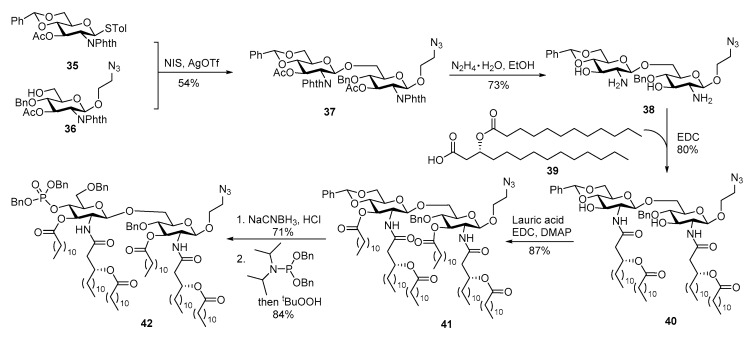
Synthesis of *N. meningitidis* MPLA with an azidoethyl group on the reducing-end glucosamine unit.

Inspired by the success of Pam_3_Cys conjugate vaccines, the Guo group designed and synthesized a series of oligosaccharide conjugate vaccines with MPLA as a vaccine carrier and built-in adjuvant (for lipid A synthesis, please refer to a recent review [92]). The synthesis of MPLA derived from *N. meningitidis* with symmetric lipid chain distribution and an aminoethyl group on the reducing-end GlcN unit is depicted in Scheme 8 [93]. After glycosylation of **36** with thioglycoside **35**, the designated amino and hydroxyl groups for lipidation were simultaneously exposed upon hydrazine treatment. Then, four long chain acyl groups were sequentially introduced onto the disaccharide to afford **40**. Regioselective reductive opening of the benzylidene ring exposed the 4′-hydroxyl group, which was phosphorylated to provide protected MPLA **42**.

**Scheme 9:**
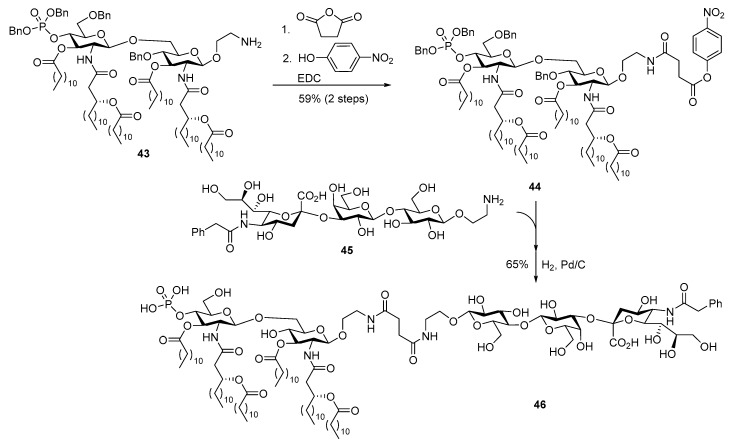
Conjugation of *N. meningitidis* MPLA with a modified GM3 antigen to form conjugate vaccine **46**.

To facilitate the coupling of carbohydrate antigens with MPLA, the azido group in **42** was reduced to free amine, which was then acylated with a bifunctional linker followed by conversion of the resultant acid into an active ester of *p*-nitrophenol (Scheme 9). Finally, the modified GM3 antigen **45** was coupled with **44** at ambient temperature, and the resulting conjugate was globally debenzylated via Pd-catalyzed hydrogenolysis to give vaccine candidate **46**. Immunological studies showed that this conjugate vaccine elicited strong immune responses without an external adjuvant, indicating its self-adjuvanting property. In addition, the conjugate induced mainly IgG3 antibody production, which suggested the elicitation of adaptive immune response with sustained immunological memory [94].

**Figure 2:**
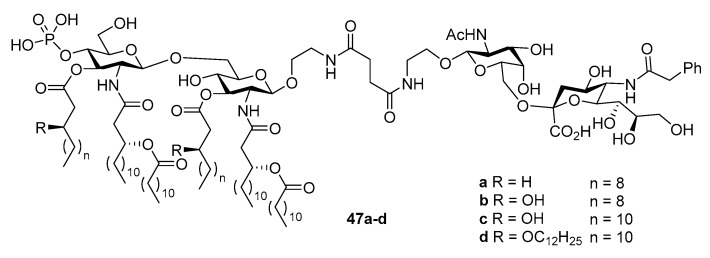
Conjugates of sTn antigen with MPLAs containing different lipid structures for SAR studies.

Encouraged by the above discovery, the Guo group conducted a series of studies to optimize MPLA structure via SAR analysis and explore this new concept with other carbohydrate antigens. As reported by Park et al. [25], five of the six lipid chains of *E. coli* lipid A are deeply buried in the hydrophobic pocket of the TLR4/MD2 heterodimer. Any change on these lipid chains is deemed to have a significant impact on the binding affinity between the two parties. Accordingly, the Guo group investigated the influence of lipid chains on the adjuvanting property of MPLA in the context of conjugate vaccines [95]. Thus, a series of MPLAs with different lipids on the 3-*O*-positions of both glucosamine units were synthesized and then coupled with sTn antigen by procedures similar to that depicted above. The resulting conjugates **47a**–**d** (Figure 2) were immunologically evaluated in mice. It was demonstrated that **47a** derived from the natural form of *N. meningitides* lipid A was the most effective in eliciting the production of both total and IgG3 antibodies. Moreover, additional or elongated lipid chains decreased the efficacy of antibody production. The results are useful for optimizing conjugate vaccine designs. 

**Scheme 10:**
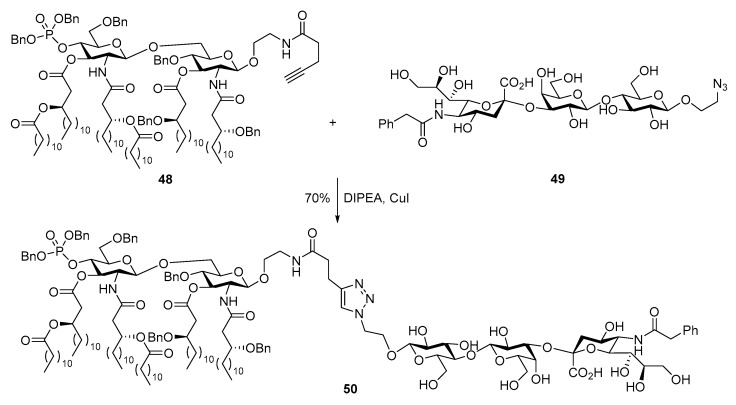
Conjugation of *E. coli* MPLA **48** with a modified GM3 antigen by click reaction.

MPLA from *E coli* lipid A, e.g., **48** (Scheme 10), has asymmetrically distributed lipid chains on each monosaccharide unit. Different from aforementioned synthesis, a highly convergent strategy featuring late stage coupling of two monosaccharides having preinstalled lipid chains was adopted for the synthesis of MPLA **29** by the Guo group [96]. This MPLA was furnished with an alkyne group at the reducing terminus, which served as the linker for direct coupling with a modified GM3 antigen **49** that had an azidoethyl group at the reducing end by click reaction. Under catalysis of copper (I) iodide, the two moieties were conjugated via a triazole linkage in a good yield (70%). 

**Scheme 11:**
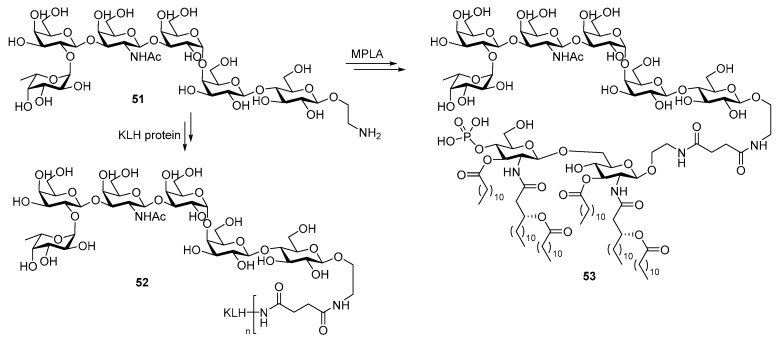
Conjugation of globo H antigen with KLH and MPLA to formulate conjugate vaccines **52** and **53**.

Globo H is a carbohydrate antigen found on multiple epithelial tumors including breast cancer, lung, colon and prostate cancers. Its conjugate with the KLH protein carrier showed potent immunogenicity against breast cancer and has reached phase III clinical trial [97]. In 2015, the Guo group synthesized the conjugate **51** of globo H with optimized *N. meningitidis* MPLA (Scheme 11) and compared it with the KLH-globo H conjugate **52** [98]. 

**Figure 3:**
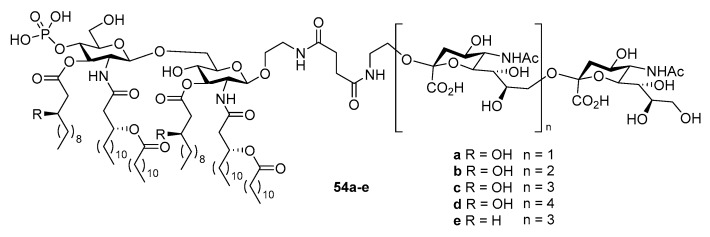
Conjugates of *N. meningitidis* MPLA with polysialic acids as group C meningitis vaccine candidates.

The same linker was used for both KLH and MPLA conjugates to ensure unbiased comparison. Overall, the MPLA conjugate was found to induce IgG antibody titers 2-fold higher than the KLH conjugate. Moreover, the immune response towards MPLA-globo H conjugate was much faster. Considering that the failure of KLH-sTn vaccine in phase III clinical trials was ascribed to slow immune response that was overwhelmed by cancer progression [99], fast immune response may be one of the desirable features of the new cancer vaccine. A series of α-2,9-oligosialic acids varying in chain length were also conjugated with MPLA, and the resultant conjugates **54a**–**e** (Figure 3) as vaccine candidates against group C meningitis were compared to the corresponding KLH conjugates [100]. 

Both types of glycoconjugates showed comparable and robust activities to elicit IgG antibody induction. Further in-depth SAR study of the MPLA conjugates revealed that the hydroxyl group on the acyl lipid chains of MPLA was important for the vaccine, since **54c** elicited stronger immune response than **54e**, which were only different in the lipid structure. It was also concluded that the overall immunogenity of these conjugate vaccines decreased with elongation of the oligosialic acid chain and higher antibody titers were observed with **54a** than all other tested conjugates. 

**Scheme 12:**
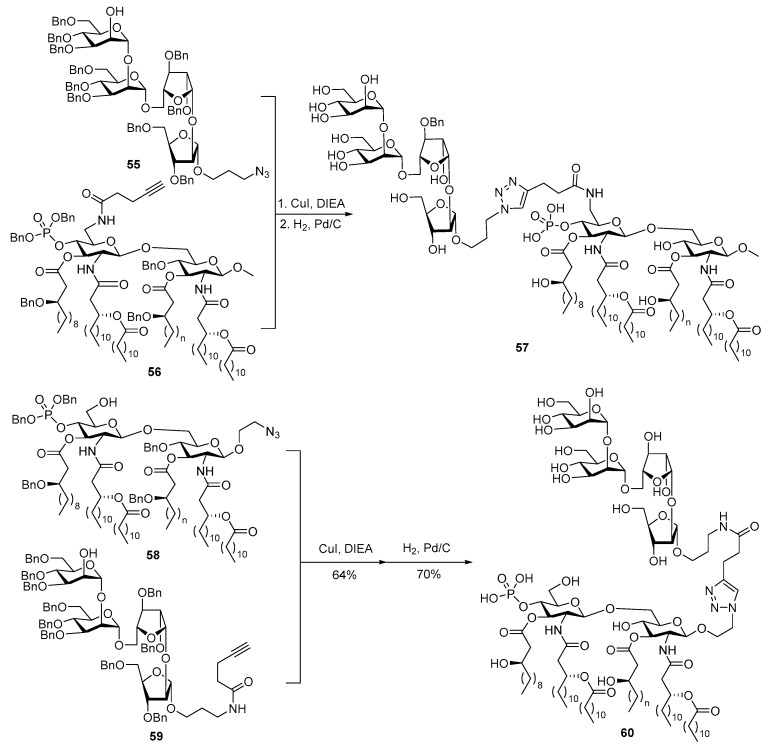
Conjugation of an upstream LAM oligosaccharide with MPLA at different positions.

In natural LPSs, the polysaccharide chain is invariably linked to the 6’-*O*-position of lipid A. To avoid any potential influence of carbohydrate antigens attached to the MPLA disaccharide reducing end on the binding between MPLA and TLR4/MD-2 heterodimer, the Guo group also synthetized a MPLA derivative with modified 6’-*O*-position **56** (Scheme 12) and examined its activity as a vaccine carrier and built-in adjuvant [101]. In this context, **56** was conjugated with **55**, which is the non-reducing end cap oligosaccharide of lipoarabinomannan (LAM), a cell surface lipopolysaccharide of *Mycobacterium tuberculosis* essential for mycobacterial infections, by click reaction. The resultant conjugate **57** was compared immunologically with conjugate **60** (Scheme 12) that had the same oligosaccharide antigen attached to the reducing end of the same MPLA. Immunological studies showed that although both conjugates induced strong IgG antibody responses, the 6′-*O*-linked MPLA conjugate **57** elicited significantly higher titers of both total antibodies and IgG antibodies as compared to the 1-*O*-linked analogue **60**. This may be attributed to the fact that the carbohydrate chain at the lipid A 6′-*O*-position in LPS is far away from the binding pocket and thus has less interactions with the TLR4/MD-2 heterodimers as demonstrated by Park et al. [25]. Consequently, having carbohydrate antigens linked to the MPLA 6′-*O*-position may be the optimal vaccine design.

**Scheme 13:**
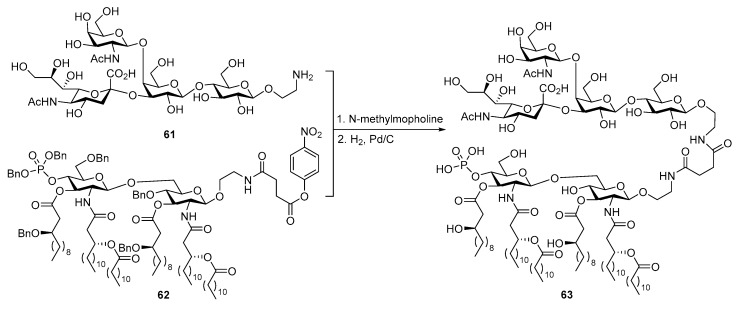
Conjugation of GM2 antigen with MPLA to form conjugate vaccine **63**.

Another TACA, the GM2 antigen, was also coupled with MPLA (Scheme 13) by a strategy similar to that used to synthesize the globo H conjugate (Scheme 11), and the resultant glycoconjugate **63** was evaluated [102]. Once again, immunological studies demonstrated the self-adjuvanting property of **63** as a vaccine candidate, since it elicited strong immune responses in mouse without combined usage with an external adjuvant, and the activity of **63** to elicit immune responses was only marginally weaker than that of the KLH-GM2 conjugate. More significantly, the antisera induced by glycoconjugate **63** showed antibody-mediated complement-dependent cytotoxicities to breast cancer cell line MCF-7, indicating the potential of **63** as a therapeutic cancer vaccine.

**Scheme 14:**
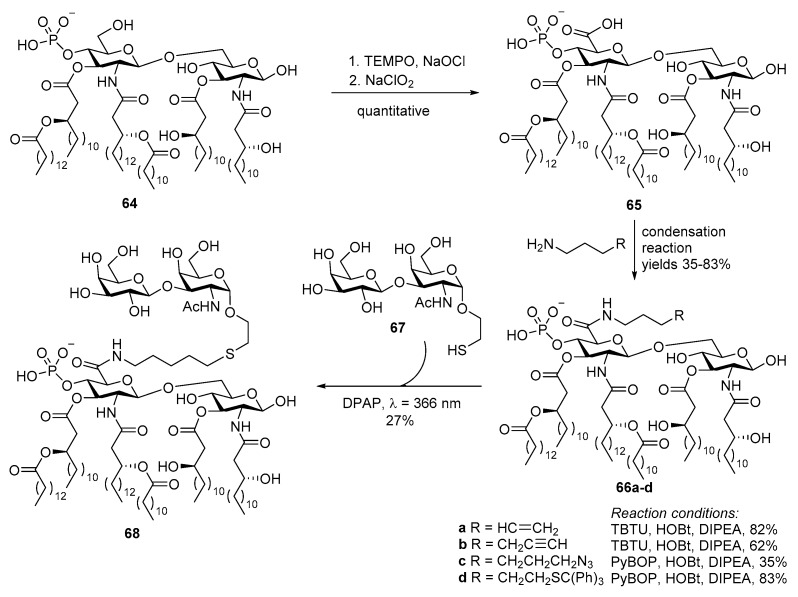
Synthesis of a MPLA derivative **64** from naturally derived MPLA and its subsequent conjugation with the TF antigen.

Due to the structural complexity of MPLA, the scale-up of its chemical synthesis is a challenge. To overcome this problem, the Bedini group took a different approach to obtain selectively activated MPLA derivatives via regioselective modification of MPLA derived from fermentation [103]. This semisynthetic method started with selective oxidation of the 6′-hydroxyl in **64** (Scheme 14). The resulting 6’-carboxylic acid derivative was then coupled with linkers bearing various functionalities, including alkene, alkyne and azide, via an amido bond. This allowed for MPLA conjugation with carbohydrate antigens by diverse coupling methods. Eventually, TF antigen with a thiol group **67** was attached to the alkene group in **66a** in presence of a photoinitiator under UV light. Immunological studies showed that the resulting conjugate **68** could successfully induce cytokine production [104]. 

### 3.4. Conjugate Vaccines with MPLA Mimics as Carrier Molecules or Built-In Adjuvants

**Scheme 15:**
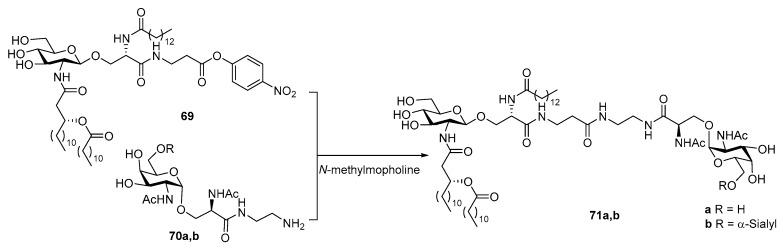
Conjugation of Tn and sTn antigens with MPLA mimic **69**.

Long before the total synthesis of natural lipid A and MPLA was pursued, researchers had foreseen the potential of lipid A in conjugate vaccine development from its success as external adjuvants. However, limited by the resources, they had to turn their attention to partial structures of lipid A. For example, in 1997, Achiwa et al. coupled Tn and sTn antigens to a partial structure of lipid A without the phosphoryl moiety [105]. They also synthesized a lipid A mimic 69 using l-serine to replace d-glucosamine (Scheme 15), which proved to be a potent mitogen [106]. This lipid A mimic was then coupled with Tn or sTn antigen bearing a diamine spacer to give conjugate **71**. Unfortunately, no immunological data was given regarding these conjugates.

**Scheme 16:**
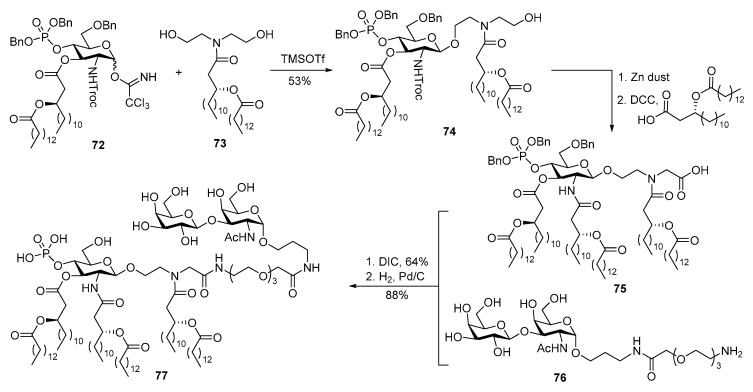
Conjugation of TF antigen with MPLA mimic **75** to prepare conjugate vaccine **77**.

As a part of their efforts to simplify the structure of lipid A while maintaining its biological activities, the Jiang group designed and synthesized a series of mimics of *E. coli* MPLA. By screening their activities to elicit ICAM-1 and cytokine production in human THP-1 cells, **75** (Scheme 16) was found to be a potent immunostimulator [107]. TF antigen was covalently linked to this MPLA mimic via a linear spacer and amide bonds, followed by deprotection under hydrogenolysis conditions to afford conjugate vaccine **77**, setting the stage for subsequent immunological studies to be reported later [108]. 

Compared to lipopeptides, lipooligosaccharides were less exploited as vaccine carriers with only a few successes exemplified by MPLA. However, it is noteworthy that even without a helper T epitope, MPLA conjugates can induce high titers of IgG antibodies, indicating the stronger immunostimulating activity of MPLA than lipopeptides such as Pam_2_Cys/Pam_3_Cys. It can be envisioned that, by implanting the innovative strategies exploited in Pam_2_Cys/Pam_3_Cys conjugates, the advantages of MPLA conjugate vaccines will be further explored in the future.

## 4. Other TLR Ligand Conjugates

Although less studied, other TLR ligands such as polyinosinic:polycytidylic acid (poly I:C, a RNA analog that activates TLR3) [109] and unmethylated oligodeoxy-nucleotides (CpG ODN) have been used together with MUC1 antigen. But due to their intrinsically poor drug properties and difficulties in characterizing the conjugates, utilization of DNA/RNA as built-in adjuvants is less attractive to researchers and only a few reports on their conjugation with glycopeptide antigens were found.

**Scheme 17:**
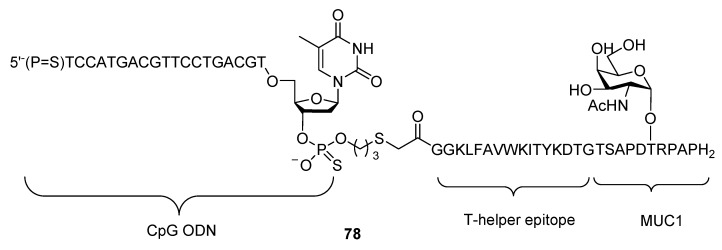
Multicomponent vaccine candidate **78** consisting of CpG ODN, T-epitope and MUC1 antigen.

A multicomponent vaccine candidate comprised of CpG ODN, T-epitope and MUC1 antigen was first designed and synthesized by Boons et al. (Scheme 17, compound **78**) [110] However, subsequent immunological study revealed that this vaccine candidate was less potent than Pam_3_CSK_4_ conjugates. Trials of incorporating CpG ODN by electrostatic interactions with T-epitope and MUC1 antigen have also been reported [111], but cannot be categorized as conjugate, therefore will not be elaborated in this review. 

## 5. Conclusions

Melding two or more moieties with diverse functions into one multi-tasking conjugate to modulate the human immune system for fighting against cancer or infectious diseases is one of the most promising therapeutic strategies. Compared to well-established protein carriers for conjugate vaccines, TLR ligands such as lipopeptides and MPLA derivatives as vaccine carriers are still at their early stage. However, since their interactions with TLRs are well studied and understood, the prosperity of using TLR ligands for conjugate vaccine development can be envisioned, especially because these molecules are not only vaccine carriers but also have adjuvant property to generate self-adjuvanting conjugate vaccines. As a critical immunostimulatory component in all types of vaccine formulations, adjuvants are expected to stimulate T cell-mediated immune responses and antibody class switching for long-term immunity. In this respect, TLR ligands are ideal candidates as they help not only elicit immune responses but also switch innate immune responses to carbohydrate antigens to adaptive responses, allowing for robust and memorable immunity. 

Preliminary data of antibody induction by conjugate vaccines derived from TLR ligands, especially MPLA, have shown their unquestionable potential. These results, together with the long-lasting studies on MPLA as an external vaccine adjuvant, suggest very promising outlook of its application as a carrier molecule in conjugate vaccine development. Currently, one of the major challenges in this field is how to obtain sufficient quantities of the vaccine for clinical studies, as the structures of lipopeptides and especially MPLA are complex and their synthesis and their conjugation with target antigens are not trivial. Semi-total synthesis and APGs synthesis are making significant input in solving this problem. The second challenge is how to reach the full potential of each moiety in a multi-component vaccine construct by rational considerations in vaccine design. As case study of vaccines compiling and crystal structures of TLR-ligand complexes elucidated, better vaccine designs may be in our reach through SAR analysis and molecular modeling. Furthermore, as suggested by the Pam_3_Cys conjugates, the immunogenecity of conjugate vaccines can be highly dependent on the conjugation strategy [12]. Therefore, after structural optimization of MPLA, efforts aiming at further improvement of the properties of its conjugates should be focused on the ligation strategies and antigen presentation patterns. For example, multivalent vaccines may help improve the presentation of antigens to APCs, whereas hydrophilic linkers may help improve their pharmacologic properties.
